# Association Between *H. pylori* Infection and Colorectal Polyps: A Meta-Analysis of Observational Studies

**DOI:** 10.3389/fmed.2021.706036

**Published:** 2022-01-18

**Authors:** Depeng Lu, Mingyu Wang, Xiquan Ke, Qiangwu Wang, Jianchao Wang, Dapeng Li, Meng Wang, Qizhi Wang

**Affiliations:** ^1^Department of Gastroenterology, The First Affiliated Hospital of Bengbu Medical College, Bengbu, China; ^2^Department of Oncology, The First Affiliated Hospital of Bengbu Medical College, Bengbu, China

**Keywords:** *H. pylori* infection, colorectal polyps, adenomatous polyps, hyperplastic polyps, meta-analysis

## Abstract

**Background:**

It has been suggested that *Helicobacter pylori* (*H. pylori*) infection is associated with hypergastrinemia and proliferation of colorectal mucosa via direct stimulation, dysbiosis of the gut microbiome, and changes in the gut microbiome, all of which may lead to the formation of colorectal polyps. However, the consensus remains lacking regarding whether *H. pylori* infection is independently associated with colorectal polyps and whether the association differs according to histological type of colorectal polyps. To summarize the current evidence regarding the relationship between *H. pylori* infection and colorectal polyps, we conducted a meta-analysis of related observational studies according to the histological types of colorectal polyps.

**Methods:**

Observational studies investigating the association between *H. pylori* infection and colorectal polyps using multivariate analyses were included by search of PubMed, Embase, and Web of Science. A random-effects model was adopted to combine the results.

**Results:**

Seventeen studies that include 322,395 participants were analyzed. It was shown that *H. pylori* infection was independently associated with overall colorectal polyps (odds ratio [OR]: 1.67, 95% CI: 1.24–2.24, *p* < 0.001; *I*^2^ = 73%). According to the histological type of colorectal polyps, *H. pylori* infection was independently associated with adenomatous polyps (APs; OR: 1.71, 95% CI: 1.47–1.99, *p* < 0.001; *I*^2^ = 86%), advanced APs (OR: 2.06, 95% CI: 1.56–2.73, *p* < 0.001; *I*^2^ = 0%), and hyperplastic polyps (HPs; OR: 1.54, 95% CI: 1.02–2.30, *p* = 0.04; *I*^2^ = 78%). Evidence based on only one study showed that *H. pylori* infection was not associated with sessile serrated polyps (SSPs; OR: 1.00, 95% CI: 0.93–1.07, *p* = 0.99).

**Conclusions:**

Current evidence from case-control and cross-sectional studies suggested that *H. pylori* infection was independently associated with colorectal APs, advanced APs, and HPs, but not with SSPs. These findings suggested *H. pylori* infection may be a possible risk factor of colorectal polyp, which is important for the prevention of colorectal polyp in the adult population.

## Background

Colorectal polyps refer to protuberance extending from the normally flat colorectal mucosa into the lumen. Clinically, colorectal polyps could be classified based on their histological features and susceptibility to malignant transformation (1). Generally, colorectal polyps could be classified according to the histological types into hyperplastic polyps (HPs), adenomatous polyps (APs), and sessile serrated polyps (SSPs) (2). Among them, APs and some subtypes of HP have been considered as precancerous lesions for colorectal cancer (CRC) (3). As for SSP, the risk factors and epidemiological features are still to be determined (4). Since CRC remains one of the most prevalent malignancies all over the world and the potential role of neoplastic polyps as precancerous lesions (5), it is important to determine the risk factors for colorectal polyps according to the histological features.

*Helicobacter pylori* (*H. pylori*) is a common gram-negative bacterium that dwells on the gastric mucosa and can secrete urea enzymes, vacuoles toxins, and cytotoxin-related genes (6, 7). It has been indicated in previous studies that the prevalence of *H. pylori* infection could be more than 50% in the general population, which has been recognized as the culprit of chronic gastritis, gastric ulcers, and gastric cancer (8, 9). Besides, it has been confirmed that *H. pylori* infection confers a 1.2–1.6 times greater risk of CRC (10). It has been suggested in preclinical studies that chronic *H. pylori* infection may cause hypergastrinemia and proliferation of colorectal mucosa *via* direct stimulation, dysbiosis of the gut microbiome, and changes in the gut microbiome, all of which may lead to the formation of colorectal polyps (10–12). Therefore, it could be hypothesized that *H. pylori* infection may be a risk factor for colorectal polyps. Accordingly, accumulating studies have been performed to evaluate the potential association between *H. pylori* infection and colorectal polyps (13–29), whereas the results of these studies are not always consistent. Although a few meta-analyses have shown that *H. pylori* infection may be associated with the prevalence of colorectal polyps (10, 30–32), particularly of AP (18, 33, 34), analyses according to the histological types of colorectal polyps were rarely performed. Besides, these meta-analyses generally included data derived from univariate analysis (10, 18, 30–34). Moreover, it remains unknown whether the association between *H. pylori* infection and colorectal polyps remains after adjustment of potential confounding factors, such as age and sex (35). Accordingly, a meta-analysis summarizing the current evidence for the association between *H. pylori* infection and the prevalence of colorectal polyps is needed. Therefore, in this study, we aimed to perfume a meta-analysis to systematically evaluate the relationship between *H. pylori* infection and colorectal polyps. Besides, the associations were analyzed according to the different histological types of colorectal polyps in this study. These findings are expected to provide theoretical evidence of *H. pylori* infection as a possible risk factor of the colorectal polyp and may be important for the prevention of colorectal polyp in the adult population.

## Methods

The Meta-analysis of Observational Studies in Epidemiology (MOOSE) guideline (36) and Cochrane's Handbook (37) were followed in this study. The protocol of the meta-analysis was not prospectively registered.

### Literature Search

The electronic databases of PubMed, Embase, and Web of Science databases were searched on February 26, 2021, with a keyword-based search strategy as (“Helicobacter pylori” or “Campylobacter pylori” OR “H. pylori” OR “HP” OR “Helicobacter” OR “Helicobacter species” OR “Helicobacter sp.” OR “Helicobacter genus” OR “Campylobacter” OR “Campylobacter infection” OR “Campylobacteriosis” OR “Helicobacter pylori infection” OR “Helicobacter infection” OR “pylori” OR “enterohepatic Helicobacter spp.” OR “Campylobacter spp.”) AND (“polyp” OR “polyps” OR “polypoid-lesion”) AND (“colon” OR “colorectal” OR “colonic” OR “rectum” OR “rectal” OR “colonic-neoplasm” OR “Intestine polyp”). The keyword-based literature search strategy has been well used in previous meta-analyses and may be more sensible for the identification of related studies. Only studies reported in English were considered. References of related articles or reviews were also analyzed.

### Study Identification

Studies that fulfilled these criteria were used: (1) observational studies published as full-length papers; (2) included adult population; (3) evaluated the association between *H. pylori* infection and colorectal polyps, such as overall colorectal polyps or colorectal polyps according to histological types, i.e., AP, advanced AP, HP, and SSP; and (4) reported odds ratios (ORs) for the above associations after adjusting for multiple confounding factors (at least for age and sex). Diagnostic methods for *H. pylori* infection were in accordance with the strategies applied among the included studies. Determination of overall colorectal polyps and their histological types were based on histological diagnosis with specimens obtained during colonoscopy examination. Reviews, preclinical studies, studies with univariate analysis, and irrelevant studies were not included.

### Data Extracting and Quality Evaluation

Two authors implemented database search, data extraction, and study quality assessment separately. If disagreements occurred, they were discussed with the corresponding author. These data were recorded: (1) author, study year, and study design; (2) participant characteristics, such as a number of participants included, mean age, and sex; (3) methods for the diagnosis of *H. pylori* infection and numbers of participants with *H. pylori* infection; (4) colorectal polyp outcomes reported and numbers of patients with each outcome; and (5) potential confounding factors adjusted in the multivariate analyses. The Newcastle-Ottawa Scale (NOS) (38) was used for study quality evaluation. This scale is rated from 1 to 9 stars and reflected the quality of the study by aspects of participant selection, comparability between groups, and outcome validation.

### Statistical Analyses

Odds ratio and the corresponding 95% CIs were extracted for every included study. Then, SEs of ORs were estimated from the 95% CIs or values of *p*. For normalization of their distribution, ORs were logarithmically transformed (37) and combined. Heterogeneity within the included cohort studies was tested via Cochrane's Q test and the estimation of *I*^2^ statistics (39). An *I*^2^ > 50% suggests a significant level of heterogeneity. A random-effects model was chosen to combine the ORs by incorporating the potential heterogeneity within studies (37). Sensitivity analyses by sequentially excluding either of the included studies were conducted to clarify the influence of a certain study on the overall results (40). Funnel plots were constructed and were used for the assessment of publication bias (41), which could be further validated by the Egger's regression asymmetry test. The RevMan (Version 5.1; Cochrane Collaboration, Oxford, UK) and STATA software were involved for statistical analyses.

## Results

### Database Search

**Figure 1:**
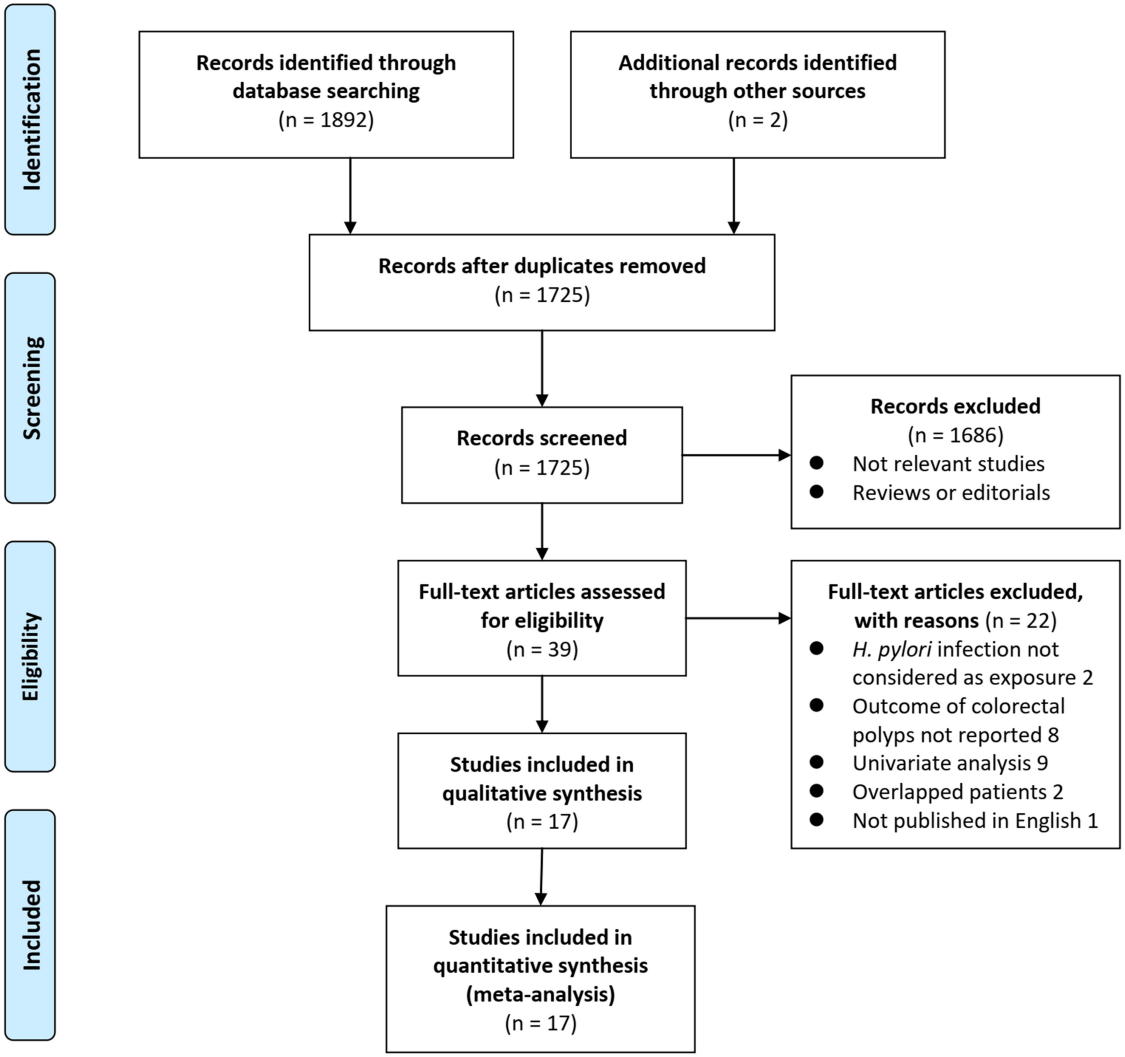
Scheme of study inclusion.

Details of the database search are shown in Figure 1. The first-step database search retrieved 1,725 articles after duplicated studies were excluded. Among them, 1,686 studies were further excluded because they were not related to the purpose of the meta-analysis based on titles and abstracts. Then, for the remaining 39 studies evaluated by full-text reading, 22 were not included for the reasons which are presented in Figure 1, which resulted in 17 studies with multivariate analyses finally included in the meta-analysis (13–29).

### Study Characteristics

Characteristics of the included studies are shown in Table 1. Overall, 17 studies, i.e., five case-control studies (13, 14, 17, 25, 27) and 12 cross-sectional studies (15, 16, 18–24, 26, 28, 29), with 322,395 participants were analyzed in the meta-analysis. The crude prevalence rates of any colorectal polyps in *H. pylori* infection and un-infection groups were 23.9% (8153/34088) and 15.0% (43170/288307), respectively. These studies were published between 1999 and 2020. Multiple methods were used for the diagnosis of *H. pylori* infection, such as serology test, ^13^C-Urea breath test, rapid urease test, and histological diagnosis with gastric specimens. A total of 34,088 participants had *H. pylori* infection (10.6%). Outcomes of overall colorectal polyps were reported in five studies (14, 20, 22, 25, 28), AP in 15 studies (13, 15–24, 26–29), advanced AP in three studies (18, 22, 23), HP in three studies (22, 27, 28), and SSP in one study (27). Age, sex, social class, body mass index (BMI), smoking, alcohol drinking, and other potential confounding factors, such as parameters of lipids and glycemic metabolism, were adjusted to a varying degree when the associations between *H. pylori* infection, and colorectal polyps were reported. The quality of these studies was good, evidenced by six to nine points of the NOS scores (Table 2).

**Table 1:** Characteristics of the included studies.

**References**	**Country**	**Design**	**Sample size**	**Mean age**	**Male**	**Diagnosis of HP infection**	**No. of patients with *H. pylori* infection**	**Outcomes reported (n)**	**Variables adjusted**
				**years**	**%**				
Breuer et al. (13)	Germany	CC	178	61.9	51.7	Serology	117	AP (89)	Age, sex, dietary factor, and BMI
Siddheshwar et al. (14)	UK	CC	236	62.5	44.9	Serology	146	Colorectal polyps (57)	Age, sex, and social class
Fujimori et al. (15)	Japan	CS	669	70.3	60.9	UBT, and RUT or histological diagnosis with gastric specimens	527	AP (327)	Age and sex
Bae et al. (16)	Korea	CS	346	54.1	73	UBT, and RUT or histological diagnosis with gastric specimens	204	AP (148)	Age, sex, and gastric dysplasia
Inoue et al. (17)	Japan	CC	478	49.5	100	Serology	368	AP (239)	Age, sex, current smoking, and TC
Hong et al. (18)	Korea	CS	2,195	49.3	61.6	Serology	1,253	AP (506), and advanced AP (103)	Age, sex, smoking, alcohol consumption, family history of CRC, and regular use of aspirin
Nam et al. (19)	Korea	CS	597	56.2	65.4	Serology	335	AP (118)	Age, sex, BMI, HbA1c and TC
Brim et al. (20)	USA	CS	1,256	57	34	Serology	366	Colorectal polyps (456), and AP (300)	Age, sex, chronic active gastritis, and baseline high risk
Patel et al. (21)	USA	CS	799	54.8	52.2	Histological diagnosis with gastric specimens	236	AP (140)	Age, sex, BMI, race, alcohol, and tobacco
Tongtawee et al. (22)	Thailand	CS	303	NR	38.3	Histological and RUT diagnosis with gastric specimens	151	Colorectal polyps (77), HP (42), AP (35), and advanced AP (13)	Age, sex, and chronic active gastritis
Nam et al. (23)	Korea	CS	4,446	NR	55.1	RUT with gastric biopsy specimen	2,246	AP (1245), and advanced AP (118)	Age, sex, BMI, educational background, smoking status, alcohol consumption, and family history of CRC
Chen et al. (24)	China	CS	1,375	53.9	NR	UBT	583	AP (180)	Age, sex, WC, BMI, SBP, DBP, TC,TG, LDL-C, and HDLC
Huang et al. (25)	China	CC	493	47.8	75.5	UBT	306	Colorectal polyps (159)	Age, sex, BMI, and family history of cancer
Yang and Yang (26)	China	CS	166	53.8	52.4	UBT	68	AP (66)	Age, sex, TC, TG, hypertension, and FPG
Sonnenberg et al. (27)	USA	CC	302,061	56.5	44.3	Histological diagnosis with gastric specimens	23,995	HP (43329), AP (97777), and SSP (14436)	Age and sex
Zhao et al. (29)	China	CS	563	55.7	65.4	UBT	163	AP (315)	Age, sex, and BMI
Wang et al. (28)	China	CS	6,234	49.2	56.8	UBT	3,024	Colorectal polyps (3872), HP (2284), and AP (1588)	Age and sex

*CC, case-control study; CS, cross-sectional study; NR, not reported; UBT, ^13^C-Urea breath test; RUT, rapid urease test; AP, adenomatous polyps; SSP, sessile serrated polyps; HP, hyperplastic polyps; H. pylori, helicobacter pylori; BMI, body mass index; TC, total cholesterol; CRC, colorectal cancer; HbA1c, glycated hemoglobin; WC, waist circumference; SBP, systolic blood pressure; DBP, diastolic blood pressure; TG, triglyceride; LDL-C, low-density lipoprotein cholesterol; HDL-C, high-density cholesterol; FPG, fasting plasma glucose*.

**Table 2:** Details of study quality evaluation *via* the NOS.

**References**	**Adequate definition of cases**	**Representativeness of cases**	**Selection of controls**	**Definition of controls**	**Adjustment of age and sex**	**Adjustment of other confounding factors**	**Ascertainment of exposure**	**Same method of ascertainment for cases and controls**	**Non-response rate**	**Total**
Breuer et al. (13)	1	0	1	1	1	0	1	1	1	7
Siddheshwar et al. (14)	1	1	1	1	1	0	1	1	1	8
Fujimori et al. (15)	1	1	1	0	1	0	1	1	1	7
Bae et al. (16)	1	0	0	1	1	0	1	1	1	6
Inoue et al. (17)	1	0	1	1	1	1	1	1	1	8
Hong et al. (18)	1	1	1	1	1	1	1	1	1	9
Nam et al. (19)	1	0	1	1	1	1	1	1	1	8
Brim et al. (20)	1	1	1	1	1	1	1	1	1	9
Patel et al. (21)	1	0	1	1	1	1	1	1	1	8
Tongtawee et al. (22)	1	1	1	1	1	0	1	1	1	8
Nam et al. (23)	1	1	1	1	1	1	1	1	1	9
Chen et al. (24)	1	0	1	1	1	1	1	1	1	8
Huang et al. (25)	1	1	1	1	1	1	1	1	1	9
Yang and Yang (26)	1	0	1	1	1	1	1	1	1	8
Sonnenberg et al. (27)	1	1	1	1	1	0	1	1	1	8
Zhao et al. (29)	1	0	1	1	1	0	1	1	1	7
Wang et al. (28)	1	1	1	1	1	0	1	1	1	8

*NOS, the newcastle-ottawa scale*.

### Association Between *H. pylori* Infection and Colorectal Polyps

**Figure 2:**
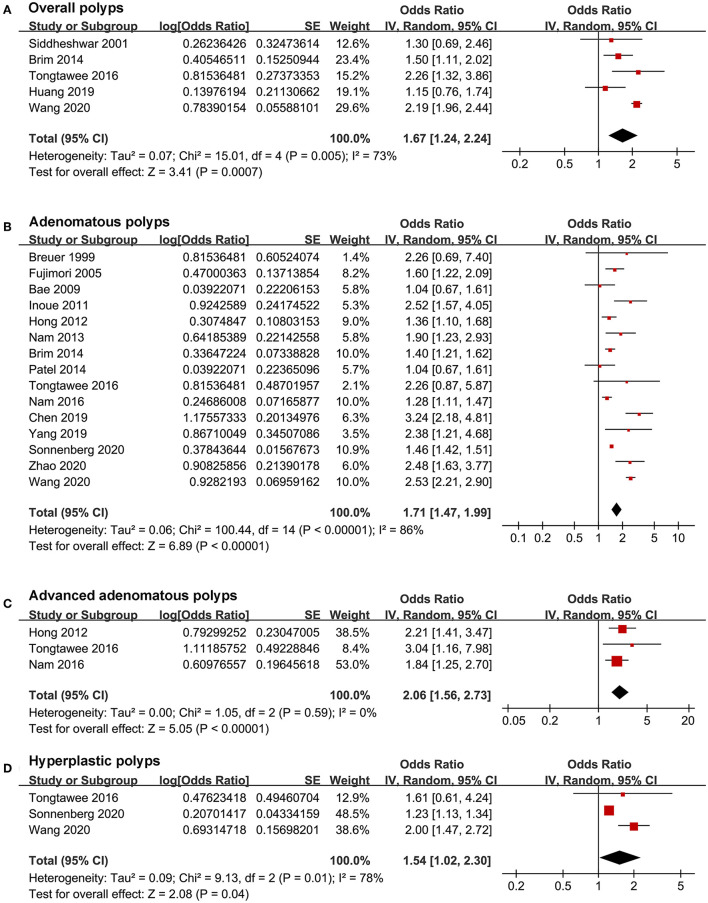
Forest plots for the meta-analysis concerning the association between *H. pylori* infection and colorectal polyps; **(A)** the outcome of overall colorectal polyps; **(B)** the outcome of adenomatous polyps; **(C)** the outcome of advanced adenomatous polyps; and **(D)** the outcome of hyperplastic polyps.

Pooled results with a random-effects model showed that *H. pylori* infection was independently associated with overall colorectal polyps (OR: 1.67, 95% CI: 1.24–2.24, *p* < 0.001; *I*^2^ = 73%; Figure 2A). According to the histological type of colorectal polyps, *H. pylori* infection was independently associated with AP (OR: 1.71, 95% CI: 1.47–1.99, *p* < 0.001; *I*^2^ = 86%; Figure 2B), advanced AP (OR: 2.06, 95% CI: 1.56–2.73, *p* < 0.001; *I*^2^ = 0%; Figure 2C), and HP (OR: 1.54, 95% CI: 1.02–2.30, *p* = 0.04; *I*^2^ = 78%; Figure 2D). Sensitivity analyses by excluding one study at a time showed consistent results (Table 3). The heterogeneity for the outcomes of overall colorectal polyps, AP, and HP was significantly reduced after excluding the study by Wang et al. (28) (*I*^2^ reduced to 25, 68, and 0% for the three outcomes of overall colorectal polyps, AP, and HP), suggesting that this study may be a major determinant of heterogeneity. Evidence based on only one dataset (27) showed that *H. pylori* infection was not associated with SSP (OR: 1.00, 95% CI: 0.93–1.07, *p* = 0.99).

**Table 3:** Results of sensitivity analysis.

**Study excluded**	**OR (95% CI)**	***I* ^2^**	***P* for Cochrane's Q test**	***P* for overall effect**
**Overall polyps**				
Siddheshwar et al. (14)	1.73 [1.26, 2.37]	77%	0.004	<0.001
Brim et al. (20)	1.71 [1.18, 2.47]	72%	0.01	0.005
Tongtawee et al. (22)	1.56 [1.10, 2.21]	80%	0.002	0.01
Huang et al. (25)	1.85 [1.42, 2.41]	61%	0.05	<0.001
Wang et al. (28)	1.47 [1.15, 1.90]	25%	0.26	0.003
**Adenomatous polyps**				
Breuer et al. (13)	1.70 [1.46, 1.99]	87%	<0.001	<0.001
Fujimori et al. (15)	1.72 [1.46, 2.03]	87%	<0.001	<0.001
Bae et al. (16)	1.76 [1.51, 2.07]	87%	<0.001	<0.001
Inoue et al. (17)	1.67 [1.43, 1.95]	86%	<0.001	<0.001
Hong et al. (18)	1.75 [1.49, 2.07]	87%	<0.001	<0.001
Nam et al. (19)	1.70 [1.45, 1.99]	87%	<0.001	<0.001
Brim et al. (20)	1.76 [1.48, 2.09]	87%	<0.001	<0.001
Patel et al. (21)	1.76 [1.51, 2.06]	87%	<0.001	<0.001
Tongtawee et al. (22)	1.70 [1.46, 1.99]	87%	<0.001	<0.001
Nam et al. (23)	1.77 [1.49, 2.11]	86%	<0.001	<0.001
Chen et al. (24)	1.63 [1.41, 1.90]	85%	<0.001	<0.001
Yang and Yang (26)	1.69 [1.45, 1.98]	87%	<0.001	<0.001
Sonnenberg et al. (27)	1.77 [1.44, 2.16]	85%	<0.001	<0.001
Zhao et al. (29)	1.67 [1.43, 1.95]	86%	<0.001	<0.001
Wang et al. (28)	1.58 [1.40, 1.79]	68%	<0.001	<0.001
**Advanced adenomatous polyps**				
Hong et al. (18)	1.97 [1.38, 2.82]	0%	0.34	<0.001
Tongtawee et al. (22)	1.99 [1.48, 2.66]	0%	0.55	<0.001
Nam et al. (23)	2.34 [1.55, 3.52]	0%	0.56	<0.001
**Hyperplasia polyp**				
Tongtawee et al. (22)	1.53 [0.95, 2.46]	89%	0.08	0.003
Sonnenberg et al. (27)	1.96 [1.46, 2.63]	0%	0.68	<0.001
Wang et al. (28)	1.23 [1.13, 1.34]	0%	0.59	<0.001

*OR, odds ratio*.

### Publication Bias

**Figure 3:**
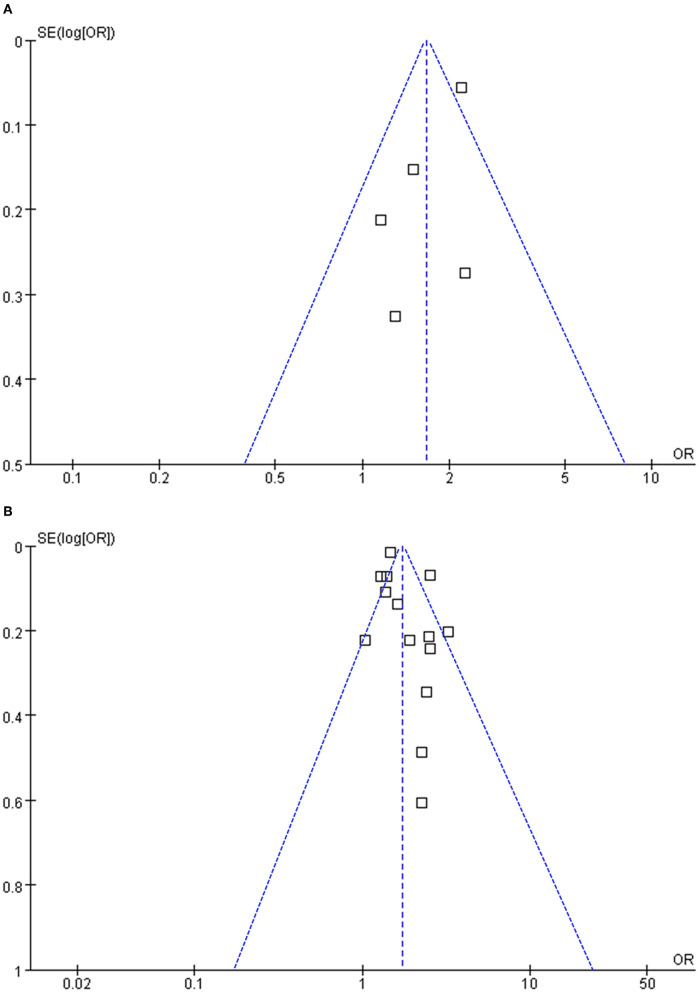
Funnel plots for the meta-analyses; **(A)** funnel plots for the meta-analysis concerning the association between *H. pylori* infection and overall colorectal polyps; and **(B)** funnel plots for the meta-analysis concerning the association between *H. pylori* infection and adenomatous polyps.

Funnel plots representing the meta-analysis of *H. pylori* infection and overall colorectal polyps and AP are shown in Figures 3A,B. The plots were symmetrical based on visual inspection, suggesting a low risk of publication bias. Egger's regression test also demonstrated a low risk of publication bias (*p* for Egger's regression tests = 0.322 and 0.128, respectively). The publication biases underlying the meta-analysis of *H. pylori* infection and advanced AP and HP were unable to determine since only three studies were included for these two outcomes.

## Discussion

In this meta-analysis, we combined the results of seventeen studies that include 322,395 participants and found that *H. pylori* infection was independently associated with colorectal AP, advanced AP, HP but not with SSP. Although large-scale prospective cohort studies are needed to determine whether *H. pylori* infection is an independent risk factor for colorectal polyps, these findings highlight the hypothesis that *H. pylori* infection is likely to be involved in the pathogenesis of colorectal polyp formation.

A few meta-analyses have been performed to evaluate the association between *H. pylori* infection and colorectal polyps. An early meta-analysis published in 2013 showed that *H. pylori* infection was associated with colorectal polyps. However, colorectal polyps outcomes with different histological types were combined and data derived from univariate analysis were pooled, which made it difficult to interpret the results (30). Since then, four other meta-analyses were performed to evaluate the association between *H. pylori* infection and AP. Although these meta-analyses consistently showed that *H. pylori* infection was associated with AP (OR: 1.49–2.05), all of these studies incorporated data with univariate analyses, which may confound the results (10, 31, 33, 34). To the best of our knowledge, only one previous meta-analysis that includes only studies of the East Asian population investigated the association between *H. pylori* infection with HP and AP separately (32). The authors showed that *H. pylori* infection was associated with AP but not with HP, but unfortunately, the results were also based on data of studies with univariate analysis (32). In view of the limitations of previous meta-analyses, we performed this study with an improvement of the study design. Firstly, only studies with data derived from multivariate analyses were included, which minimized the potential influences of confounding factors on the association between *H. pylori* infection and colorectal polyps. Secondly, the association between *H. pylori* infection and colorectal polyps was analyzed according to the histological types because of the different clinical and biological characteristics of the lesions. We found that *H. pylori* infection was independently associated with colorectal AP, advanced AP, HP but not with SSP.

The results of meta-analyses were consistent with the previous hypothesis that *H. pylori* infection plays important role in the progression of colorectal adenoma formation, advancing and malignant transformation. Several mechanisms may account for the role of *H. pylori* infection in this process. Firstly, chronic *H. pylori* infection causes hypergastrinemia, which leads to the proliferation of colorectal mucosa and susceptibility to carcinogenesis (42). Secondly, *H. pylori* infection induced gastric mucosal atrophy and decrease gastric acid secretion, which subsequently lead to dysbiosis of the gut microbiome and changes in the gut microbiome, an important pathophysiological component of colorectal mucosa carcinogenesis (43, 44). Besides, since *H. pylori* has been observed to reside in the colorectum and is associated with colorectal neoplasia, a direct carcinogenesis effect of *H. pylori* infection to colorectal mucosa may also exist (45). Future studies are needed to determine the exact mechanisms and molecular pathways involved. Moreover, data from one study showed that *H. pylori* infection was not associated with SSP, reflecting that the biological feature of the related risk factors for SSP is different from other colorectal polyps (46), such as AP. Although SSPs are much less common compared to AP, in view of the potential malignant transformative nature of SSP, more studies are needed to determine the risk factors of SSP (47). Currently, it remains unknown why *H. pylori* infection was not associated with SSP. However, previous studies showed a few epidemiological and biological differences between SSP and other forms of colorectal polyps (48). For example, SSP with dysplasia or invasive carcinoma was associated with advanced age, female sex, and proximal colon (48). However, it has been confirmed that male sex is an independent risk factor for *H. pylori* infection (49), while women are less likely to have *H. pylori* infection as compared with men. Future studies are needed to validate whether *H. pylori* infection is not associated with the formation of SSP.

This study also has limitations. Firstly, significant heterogeneity existed among the included studies. Results of sensitivity analysis showed that the heterogeneity for the outcomes of overall colorectal polyps, AP, and HP was significantly reduced after excluding the study by Wang et al. (28), suggesting that this study may be a major determinant of heterogeneity. In particular, the prevalence of colorectal polyps was highest in the HP negative group in the study by Wang et al. (28) among the included studies, suggesting that patients in this study may have a higher risk for colorectal polyps as compared to those in other studies, which lead to the significant heterogeneity. Besides, the meta-analysis was based on data from the study level but not from individual patients, which prevented further analyses on the influence of patient characteristics on the outcome, such as the ethnicity, age, sex, and comorbidities of the participants. In addition, diagnosis of *H. pylori* infection varied among the included studies, and the association between *H. pylori* infection with colorectal polyps according to the different *H. pylori* infection diagnostic strategies remains unknown. Moreover, although only studies with multivariate analyses were included, we were unable to exclude the possibility that there may be residual factors that may still confound the association. Furthermore, studies evaluating the association between *H. pylori* infection with advanced AP, HP, and SSP are limited. Therefore, the results for these outcomes should be validated in future studies. Finally, only case-control and cross-sectional studies were included, and no prospective cohort studies are available regarding the association between *H. pylori* infection and colorectal polyps. Accordingly, it is still unknown whether *H. pylori* infection is an independent risk factor for colorectal polyps.

In conclusion, this meta-analysis showed that *H. pylori* infection is independently associated with colorectal polyps, and the associations are consistent for AP, advanced AP, HP but not for SSP. Large-scale prospective cohort studies are warranted to determine whether *H. pylori* infection is an independent risk factor for colorectal polyps.

## Data Availability Statement

The original contributions presented in the study are included in the article/supplementary material, further inquiries can be directed to the corresponding author/s.

## Author Contributions

DLu and QizW designed the study. DLu and MiW performed literature search, study identification, data extraction, and study quality evaluation. DLu, XK, QiaW, JW, DLi, and MeW performed statistical analyses and interpreted the results. DLu drafted the manuscript. All authors revised the manuscript and approved the submission.

## Conflict of Interest

The authors declare that the research was conducted in the absence of any commercial or financial relationships that could be construed as a potential conflict of interest.

## Publisher's Note

All claims expressed in this article are solely those of the authors and do not necessarily represent those of their affiliated organizations, or those of the publisher, the editors and the reviewers. Any product that may be evaluated in this article, or claim that may be made by its manufacturer, is not guaranteed or endorsed by the publisher.
